# Echinococcosis of the brain, liver, and lungs: A rare case report

**DOI:** 10.1097/MD.0000000000047129

**Published:** 2026-01-02

**Authors:** Baixin Li, Zhihong Han, Chengde Ren

**Affiliations:** a Department of Urology, Affiliated Hospital of Qinghai University, Xining, China; b Emergency Internal Medicine, Qinghai Provincial People’s Hospital, Xining, China.

**Keywords:** case report, cerebral echinococcosis, hematogenous dissemination, hepatic echinococcosis, pulmonary echinococcosis

## Abstract

**Rationale:**

Echinococcosis is a zoonotic disease most commonly caused by the larval stage of *Echinococcus granulosus*. As an intermediate host, *Homo sapien*s primarily exhibits hepatic involvement. Although hepatic involvement is the typical presentation, disseminated multi-organ infection is exceedingly rare.

**Patient concerns:**

The patient was a 22-year-old Tibetan male. He experienced an epileptic seizure 1 day prior, without an apparent cause, and occasionally lost consciousness, though there was no nausea or vomiting.

**Diagnoses:**

Cerebral echinococcosis, hepatic echinococcosis, and pulmonary echinococcosis

**Interventions:**

Microsurgical excision of the hydatid cyst was performed to alleviate the intracranial pressure it exerted on the brain tissue.

**Outcomes:**

The patient experienced no obvious discomfort following the operation, and no abnormalities were detected during routine follow-ups. Consequently, the patient was discharged without complications. Currently, the patient has resumed a normal life, and ongoing treatment for liver and lung hydatid disease should continue.

**Lessons:**

We report on a rare case characterized by specific clinical manifestations. Additionally, we examine the role of blood transmission in the dissemination of the disease and evaluate the efficacy of surgical interventions.

## 1. Introduction

Echinococcosis primarily encompasses 2 severe zoonotic taeniases, namely cystic echinococcosis (CE) and alveolar echinococcosis (AE), caused by *Echinococcus granulosus* sensu lato (s.l.) and *E. multilocularis*, respectively.^[1]^ A variety of herbivorous and omnivorous animals serve as intermediate hosts for *Echinococcus* larvae. These animals become infected upon ingesting parasite eggs from contaminated food or water sources, after which the parasites develop into larvae within their internal organs. Carnivores act as the definitive hosts, harboring mature tapeworms in their intestines; infection in these carnivores occurs through the consumption of visceral organs from intermediate hosts that carry the parasites.^[2]^

This article provides a detailed description of a rare case of disseminated echinococcosis affecting the brain, liver, and lungs. While multi-organ involvement in echinococcosis is documented, the simultaneous presentation of clinically significant cysts in the brain, liver, and lungs within a single patient is exceedingly rare.^[3]^ Most literature reports on disseminated disease often describe sequential involvement or primary involvement of the liver and lungs, with secondary spread to other sites. Cases with synchronous triple-organ (cerebral, hepatic, and pulmonary) lesions at initial diagnosis are sparsely reported and pose significant diagnostic and therapeutic challenges. This case report aims to detail the unique clinical presentation, the strategic approach to managing the life-threatening cerebral lesion while coordinating care for the hepatic and pulmonary foci, and the lessons learned regarding potential hematogenous dissemination. By comparing our experience with the limited existing literature, this case adds valuable insight into the management of this complex and rare form of disseminated echinococcosis.

## 2. Case information

A 22-year-old male Tibetan patient from China presented to our hospital 1 day prior with unprovoked headache and epileptic seizures. Detailed medical history inquiry revealed that the patient resided in a pastoral area endemic for echinococcosis, where conditions such as cohabitation with definitive hosts (e.g., dogs) and potential contamination of soil and water sources by parasite eggs are common. To further confirm the diagnosis, magnetic resonance imaging (MRI) and ultrasound examinations were performed. The results indicated space-occupying lesions in the patient’s brain, liver, and lungs. Based on the medical history and imaging findings, echinococcosis was initially suspected. Physical examination showed tenderness over the left skull and concomitant hepatomegaly.

**Figure 1.**
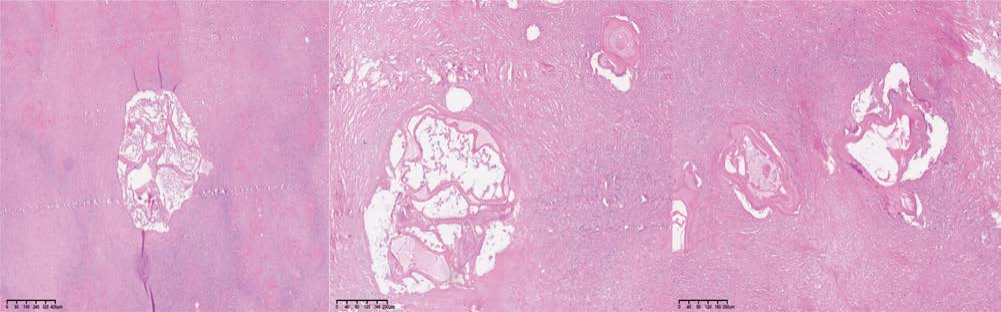
Pathological image of cerebral hydatid.

Laboratory findings were as follows: white blood cell count, 8.18 × 10⁹/L; red blood cell count, 4.75 × 10¹²/L; platelet count, 333 × 10⁹/L; neutrophil count, 5.04 × 10⁹/L; hemoglobin level, 135 g/L; D-dimer level, 0.8 mg/L; and alkaline phosphatase, 149 U/L. Serological testing revealed positive results for specific anti-echinococcosis IgG antibodies. The postoperative pathological examination confirmed cerebral vacuolar echinococcosis. Microscopic analysis of the excised cyst revealed the characteristic laminated membrane, an acellular band-like structure, accompanied by fragments of the germinal layer where protoscolices may develop. The surrounding brain tissue showed gliosis and chronic inflammatory cell infiltration. These features are diagnostic for echinococcosis (Fig. 1).

**Figure 2.**
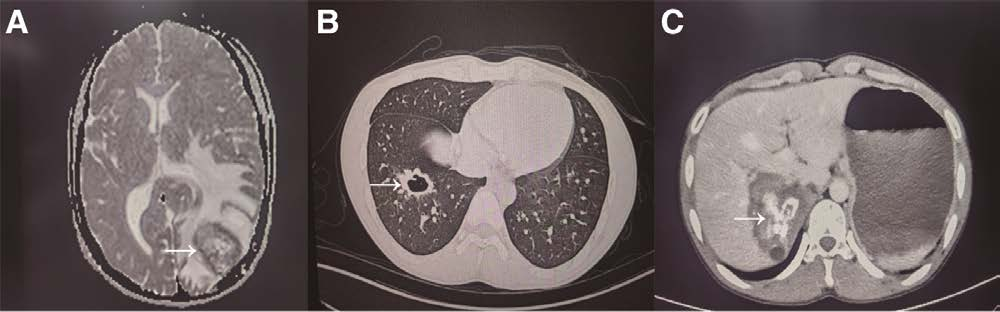
(A) MRI shows hypoperfusion within the lesion and hyperperfusion at the margin, suggesting alveolar echinococcosis (AE) (horizontal white arrow, parameters: T2-TSE; TR/TE, 3500/95 ms; slice thickness, 5 mm). (B) CT shows a lesion in the anterior basal segment of the lower lobe of the right lung with cavity formation, suggesting hepatic multilocular echinococcosis lung M (horizontal white arrow, parameters: tube voltage, 120 kV; slice thickness, 1.0 mm). (C) CT shows hepatic multilocular echinococcosis (horizontal white arrow, parameters: tube voltage, 120 kV; slice thickness, 5 mm). CT = computed tomography, MRI = magnetic resonance imaging.

The imaging findings were as follows: The head MRI revealed a single cystic lesion in the left occipital lobe (Fig. 2A); the chest CT showed solid nodules in the anterior and external basal segments of the right lower lobe (Fig. 2B); and the abdominal CT demonstrated multilocular echinococcosis in the right hepatic lobe, with involvement of the right adrenal gland (Fig. 2C).

## 3. Treatment

The patient was admitted with a headache and epileptic seizures. Imaging findings, in conjunction with the clinical presentation, led to a preliminary diagnosis of cerebral, hepatic, and pulmonary echinococcosis. Given the significant mass effect and symptoms from the cerebral cyst, and the failure of long-term oral albendazole to control the disease (posing a risk of progression), it was decided, after discussion, to perform a microsurgical resection of the supratentorial superficial lesion to alleviate intracranial pressure on the brain tissue. Under general anesthesia, the patient was placed in the right lateral decubitus position. A left occipital curvilinear skin incision was made, followed by a craniotomy centered over the lesion identified on preoperative MRI. The dura was opened in a cruciate fashion, revealing the underlying cerebral cortex. Under high-power microscopic visualization, a well – demarcated, whitish, cystic mass was found in the left occipital lobe, with minimal adhesion to the surrounding brain tissue. Critical measures to prevent cyst rupture were meticulously implemented throughout the dissection. First, the surgical field was carefully walled off with cottonoids soaked in hypertonic saline (3%) to isolate the cyst and inactivate any stray protoscoleces in case of accidental leakage. Gentle dissection was performed along the cleavage plane between the cyst wall and the neuropil. A fine - tipped suction apparatus was kept on standby while avoiding direct grasping or puncture of the cyst. The cyst was then carefully removed en bloc by leveraging the pressure difference created by irrigating the potential space between the cyst and the brain with hypertonic saline, which facilitated the cyst’s extrusion. The intact cyst was successfully removed in its entirety. The surgical bed was then copiously irrigated with hypertonic saline before standard closure.

The patient was transferred back to the ward smoothly following the surgery. Histopathological examination confirmed a definitive diagnosis of typical cerebral AE. Post-surgery, the patient was prescribed oral albendazole tablets at a dosage of 10 mg/(kg·d). Twelve days postoperation, the patient was discharged and continued with oral albendazole for 3 months. A follow-up brain CT scan 3 months later revealed no signs of echinococcosis recurrence. The pulmonary and hepatic hydatid lesions were managed with ongoing oral medication. The patient’s overall condition is currently satisfactory, with no significant discomfort. Treatment for the hepatic and pulmonary hydatid disease was recommended to continue, liver function was closely monitored throughout the treatment and remained within normal limits. There was a plan to adjust the therapy if any hepatotoxicity occurred. The timeline of key events from presentation to follow-up is summarized in Table 1.

**Table 1:** Timeline of key clinical events.

Time point	Key event	Outcome/management
Day 0 (admission)	Initial presentation with headache and epileptic seizure	Hospitalized for evaluation
Day 1	Diagnosis confirmed by MRI/CT (brain, lung, liver lesions) and positive serology	Planned for surgery
Day 5	Surgical intervention: microsurgical excision of the cerebral hydatid cyst	Surgery successful; postoperative pathology confirmed cerebral alveolar echinococcosis
Day 17 (postoperative day 12)	Discharged from hospital	Prescribed oral albendazole for 3 mo
3 mo post-discharge	Follow-up examination	Brain CT showed no recurrence; patient asymptomatic with ongoing medication for hepatic/pulmonary lesions

CT = computed tomography, MRI = magnetic resonance imaging.

## 4. Discussion

Echinococcosis, primarily caused by *E. granulosus*, exhibits distinct geographical clustering, prevalent in regions with intensive animal husbandry – including the Mediterranean coast, Middle East, South America, Oceania (sheep-farming dominant), and northwest Chinese agricultural-pastoral areas (sporadic in eastern China).^[4]^ Organ involvement is most common in the liver (60%-75%), followed by lungs (15%–20%), with renal (2%–4%), muscle/spleen/soft tissue (4% each), brain (3%), bone (2%), and other organs (1%) less frequently affected.^[5]^

This case report presents a rare instance of synchronous cerebral, hepatic, and pulmonary involvement in a young Tibetan male, who was definitively diagnosed with AE, with the brain pathology serving as the diagnostic gold standard, accompanied by hepatic and pulmonary cystic lesions radiologically suggestive of CE in the absence of molecular confirmation. While the cerebral lesion was pathologically confirmed as AE (caused by *E. multilocularis*), the cystic nature of the hepatic and pulmonary lesions on imaging is more typical of CE (caused by *E. granulosus* sensu lato). This unique triple - organ involvement presents a particular challenge for clinical diagnosis and treatment planning.

A key distinguishing feature of this case is that symptomatic cerebral lesions (presenting as epilepsy) constituted the initial clinical manifestation, whereas asymptomatic hepatic and pulmonary lesions were incidentally identified via systematic imaging studies. While disseminated echinococcosis has been previously documented in the literature, the synchronous emergence of clinically relevant lesions in the brain, liver, and lungs at initial diagnosis remains exceedingly uncommon. Most reported cases either involve isolated cerebral lesions or metachronous spread following the development of hepatic lesions.^[6]^ This unusual diagnostic sequence highlights a critical clinical pearl: in patients diagnosed with cerebral echinococcosis in endemic regions, comprehensive whole-body imaging (e.g., chest and abdominal computed tomography [CT]) is imperative, even in the absence of organ-specific symptoms. This step is essential to fully characterize the disease extent and prevent oversight of occult disseminated foci.

Concurrent involvement of the liver, lungs, and brain provides strong evidence supporting hematogenous dissemination as the primary route of spread. Plausibly, larvae originating from hepatic foci invade the hepatic venous system or lymphatic vessels, subsequently gaining access to the systemic circulation. Parasitic emboli then travel to the lungs via the right heart; should these emboli bypass the pulmonary circulation (e.g., through physiological shunts or a patent foramen ovale), they can enter the systemic arterial system and ultimately disseminate to distal organs, including the brain.^[7]^ The differential diagnosis for this case focused on cerebral space-occupying lesions, with critical distinctions needed between brain abscesses (typically associated with fever and elevated inflammatory markers), primary or metastatic brain tumors (often demonstrating irregular contrast enhancement on imaging), and neurocysticercosis. Serological positivity, the multilocular morphology of hepatic lesions, and relevant epidemiological history emerged as key differentiating factors.

The discordant pathological and imaging features in this case – with the cerebral lesion confirmed as AE while the hepatic and pulmonary lesions exhibited cystic characteristics – warrant further discussion. In the absence of molecular confirmation from the liver and lung lesions, definitive species identification for these cysts remains unconfirmed. However, several plausible explanations exist. First, although rare, co-infection with both *Echinococcus granulosus* sensu lato (causing CE) and *E. multilocularis* (causing AE) is feasible in endemic areas. A more likely explanation, consistent with the principle of parsimony, is that all lesions originated from *E. multilocularis*. The primary hepatic focus may have disseminated hematogenously. It is well recognized that metastatic AE lesions in organs such as the brain and lungs can occasionally present with a more cystic, CE-like appearance on imaging – rather than the typical infiltrative, “alveolar” pattern characteristic of hepatic AE. This atypical presentation in extrahepatic organs could be attributed to differences in host tissue responses or the stage of cyst development. Therefore, we posit that this represents a case of disseminated AE with heterogeneous morphological expression across different organs.^[8]^

Guided by the unique attributes of this case – urgent cerebral symptomatology versus occult hepatic and pulmonary lesions – a phased, multimodal, and individualized treatment approach was implemented. Priority was accorded to addressing the life-threatening symptomatic cerebral cysts: microsurgical resection was performed, supplemented by stringent anti-rupture protocols, with the dual goals of alleviating mass effect and reducing recurrence risk. For the asymptomatic hepatic and pulmonary lesions, long-term albendazole therapy was initiated, which serves as the cornerstone for controlling disseminated echinococcal foci. This strategy eschews a “one-size-fits-all” paradigm and reflects the principles of precision medicine, wherein interventions are tailored to the clinical urgency of organ involvement and the presence of symptomatology.

In summary, this case offers valuable novel insights into the diagnosis and management of disseminated echinococcosis. First, it confirms that cerebral involvement can herald the presence of occult multi-organ disease, underscoring the need for a paradigm shift toward routine whole-body imaging in such patients. Second, synchronous involvement of 3 organs suggests that the disease may exhibit greater hematogenous dissemination aggressiveness than previously appreciated. Finally, our experience validates that a phased, multimodal treatment strategy – surgical intervention for life-threatening lesions and long-term pharmacologic management for asymptomatic foci – represents an effective approach to managing these complex cases.

## 5. Conclusion

This case report describes a rare instance of echinococcosis involving concurrent involvement of the brain, liver, and lungs. Despite the significantly increased complexity of diagnosis and management due to multi-organ involvement, as well as the high risk of severe complications arising from cyst rupture during surgery for cerebral echinococcosis, the successful management of this case demonstrates that complete cyst excision aided by refined microsurgical techniques, rigorous perioperative antiparasitic drug therapy, and close collaboration among multidisciplinary teams (including neurosurgery, infectious diseases, and radiology departments) can effectively control the disease and achieve favorable neurological outcomes. This case further underscores the critical importance of complete surgical resection of cysts in the treatment of cerebral echinococcosis for reducing recurrence risk and improving patient prognosis.

## 6. Limitation

While this detailed case analysis provides important clinical insights, we recognize the need for larger prospective studies. In the future, large sample size and multi-center studies could be conducted.

## Author contributions

**Resources:** Chengde Ren.

**Supervision:** Chengde Ren.

**Writing – original draft:** Baixin Li.

**Writing – review & editing:** Zhihong Han.
